# Making Ends Meet: Chemically Mediated Circularization of Recombinant Proteins

**DOI:** 10.1002/cbic.201300105

**Published:** 2013-04-04

**Authors:** Ben Cowper, David J Craik, Derek Macmillan

**Affiliations:** [a] Department of Chemistry, University College London, Christopher Ingold Building 20 Gordon Street, London, WC1H 0AJ (UK) E-mail: d.macmillan@ucl.ac.uk; [b] Institute for Molecular Bioscience, University of Queensland Brisbane, Queensland, 4072 (Australia)

**Keywords:** acyl transfer, cyclotides, native chemical ligation, peptides, protein engineering

Circular proteins have been identified in plants (cyclotides), fungi (amatoxins/phallatoxins), bacteria (bacteriocins/pilins) and animals (defensins), where they are commonly involved in host defence against pests and pathogens.[1] Their pesticidal and antimicrobial activity, along with the high stability and bioactivity provided by a cyclic backbone, makes them valuable agricultural and pharmaceutical drug lead molecules, and necessitates the development of efficient methods for circular-protein production. Biosynthetic pathways usually involve multistep post-translational modification of propeptides by processing enzymes.[2] Direct isolation of endogenous circular protein is feasible in many instances[3] but usually requires large quantities of source biomaterial and does not lend itself to the introduction of amino acid substitutions. At present, circular proteins are most commonly obtained through use of chemical and/or biological syntheses. Established strategies for circular protein synthesis include the assembly of a linear precursor peptide through solid-phase peptide synthesis (SPPS), typically with inclusion of an N-terminal cysteine and a C-terminal thioester enabling cyclization through native chemical ligation (NCL).[4] Alternatively, recombinant circular proteins have been obtained through bacterial expression of intein fusion proteins, which afford circular proteins through expressed-protein ligation or protein trans-splicing,[5] or using sortase, a bacterial transpeptidase.[6]

A single C-terminal cysteine is capable of initiating an intramolecular N→S acyl shift in native peptide sequences.[7] This gives rise to an S-acyl intermediate that can be intercepted by an added thiol, causing cleavage of the protein backbone, liberating cysteine and yielding a C-terminal thioester. This reaction can be selective, depending on the nature of the amino acid preceding cysteine, and occurs at a significantly slower rate when cysteine is not positioned at the C terminus.[8] Furthermore the presence of cysteine at the N terminus leads to spontaneous backbone cyclization, through intramolecular transthioesterification, and S→N rearrangement forming a peptide bond (Scheme 1).[9]

**Scheme 1:**
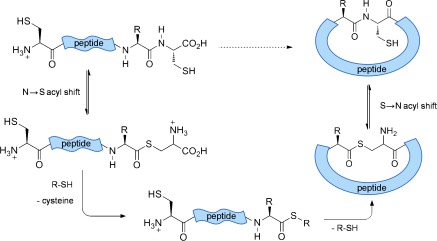
Xaa-Cys motifs undergo N→S acyl shift upon heating and the S-acyl intermediate is ultimately intercepted by an N-terminal cysteine, yielding a cyclic thioester which rearranges to form a peptide bond.

The cysteine-rich nature of many circular peptides facilitates the design of sequences containing these necessary labile sites. This strategy lends itself to the chemical synthesis of circular peptides;[9] however, the genetic encodability of the necessary labile sites should also enable application to recombinant proteins. Bacterial peptide/protein production has several advantages over chemical synthesis; providing efficient access to large polypeptides that are beyond the scope of SPPS, at relatively low cost, and facilitating the production of combinatorial peptide libraries, through straightforward DNA engineering.[10]

Here we demonstrate the applicability of this strategy to recombinant proteins using the plant cyclotide kalata B1 (KB1). Cyclotides are perhaps the most comprehensively studied family of circular proteins to date, with hundreds of members identified and numerous 3D structures available.[3] They contain 28–37 amino acids, including six conserved cysteine residues that form three disulfide bonds, giving rise to a rigid, characteristic “cyclic cystine knot” structure, which is extremely stable.[11] These cysteines aside, the cyclotide family exhibits high sequence variability, and consequently cyclotides have emerged as valuable protein engineering scaffolds for the incorporation and stabilisation of bioactive peptide sequences.[12] KB1, from the tropical herb *Oldenlandia affinis*, was the first cyclotide to be identified and is considered to be the prototypic family member.[13]

Although synthesis of a 30-residue peptide is within the bounds of SPPS, the often inefficent production of cysteine-rich peptide thioesters employing 9-fluorenylmethyloxycarbonyl (Fmoc) SPPS chemistry means that less desirable *tert*-butyloxycarbonyl (Boc) SPPS chemistry (which generally requires a potentially hazardous HF resin cleavage step) is typically employed.[14] This synthetic limitation enhances the requirement for alternative recombinant-based strategies for cyclotide production.

Bacterial expression of a recombinant kalata B1 linear precursor peptide was facilitated through fusion with an N-terminal thioredoxin (Trx) tag, yielding approximately 60 mg of purified protein per litre of cell culture. Conveniently, one of the six cysteines of wild-type KB1 is preceded by glycine. These sequential glycine and cysteine residues were therefore designated as the respective C- and N-terminal KB1 residues in the genetically encoded sequence, and a seventh cysteine was appended to the C terminus to provide a labile Gly-Cys site for N→S acyl shift. The His-tagged Trx-KB1 fusion protein was purified through immobilized Ni^2+^-affinity chromatography (Figure 1 A and Figure S1 in the Supporting Information), and the linear KB1 peptide was liberated initially through factor Xa protease (Figures S2 and S3), yielding linear KB1 with an N-terminal cysteine. Subsequently more efficient liberation was achieved by employing tobacco etch virus (TEV) protease (Figure 1 B). The desired KB1 peptide was purified through reversed phase-high performance liquid chromatography (RP-HPLC) in an unoptimised yield of 35 %, based on the Trx-fusion precursor (Figure 1 C), and characterized by mass spectrometry (Figure 1 D, left).

**Figure 1:**
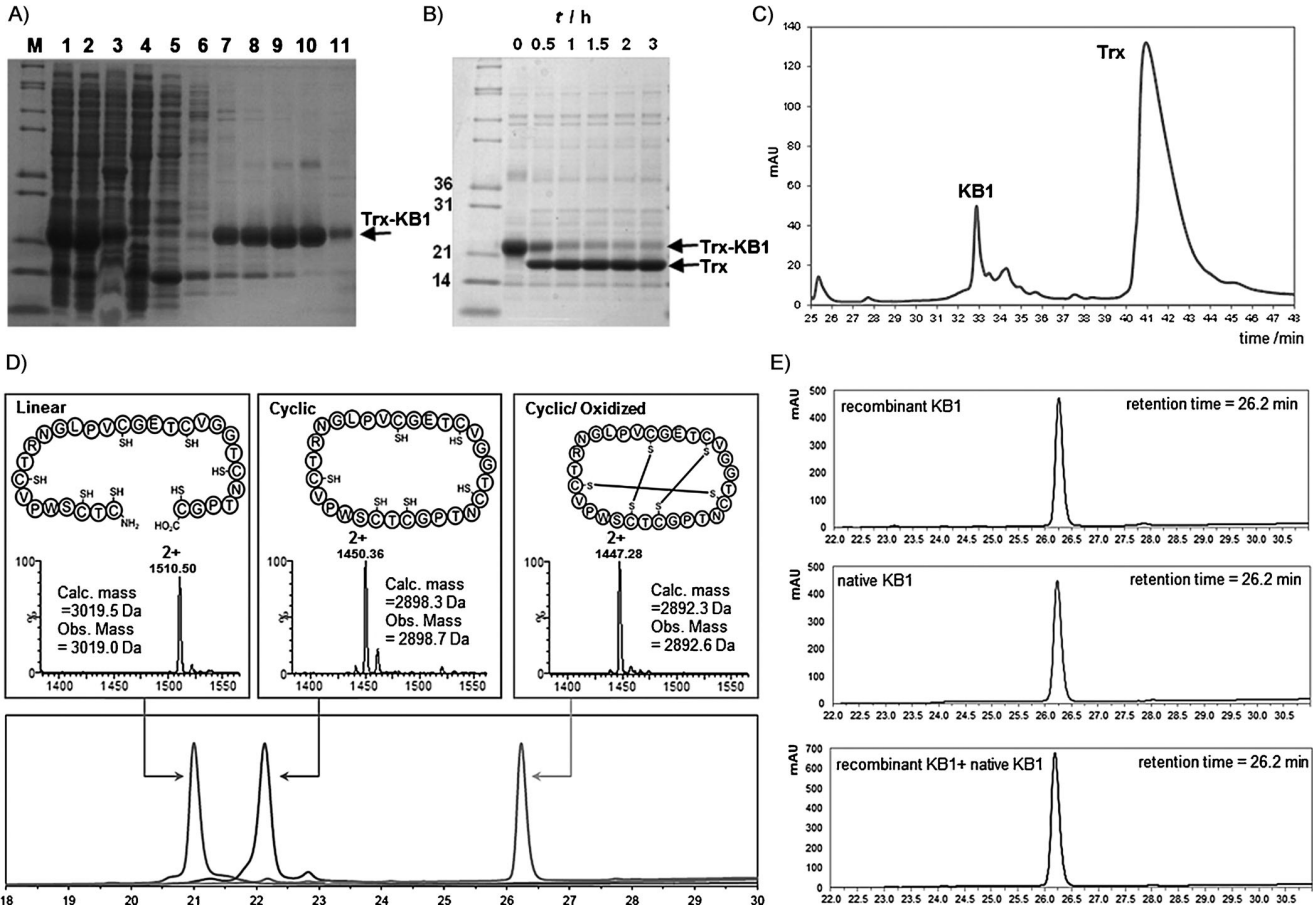
Production of KB1. A) SDS-PAGE analysis of Ni^2+^-affinity-purified Trx-KB1 fusion protein. M: molecular weight markers. Lane 1: whole-cell lysate, lane 2: soluble fraction, lane 3: insoluble fraction, lane 4: column flow-through, lane 5: column wash (5 mm imidazole), lane 6: column wash (20 mm imidazole), lanes 7–11: eluted fractions (40–500 mm imidazole). B) TEV protease digestion of the fusion protein shows accumulation of Trx (released linear KB1 is not visible on the gel). C) Preparative HPLC allows straightforward separation of the released KB1 from Trx. D) Analytical HPLC (lower panel) and MS (upper panels) characterization of purified linear, cyclic (reduced) and folded KB1 samples. E) HPLC coelution experiment: KB1 (upper panel), native KB1 (isolated from *O. affinis*, middle panel) and a 1:1 mixture of each peptide (lower panel).

Subsequent in vitro cyclization of the purified linear KB1 peptide was achieved through incubation at 45 °C in the presence of 10 % (*w*/*v*) sodium 2-mercaptoethane sulfonate (MESNa). The reaction proceeded for 48 h, after which the linear starting material was almost entirely consumed (Figure S4). The observed drop in molecular mass of approximately 121 Da in the cyclic product was consistent with the excision of the C-terminal cysteine residue and backbone cyclization (Figure 1 D, middle panel). In order to demonstrate that we had produced the correct circular framework, oxidative folding was performed, as previously described,[13] and confirmed by a further loss of six mass units (Figure 1 D, right). We also observed a characteristic increase in HPLC retention time on a reversed-phase chromatography column following cyclization and oxidation (Figure 1 D lower panel), due to the exposure of surface hydrophobic residues in the folded structure,[3] thus indicating the presence of natively folded KB1. Over 1 mg of purified folded material was obtained from 2 L cell culture. Furthermore, we carried out analytical coelution RP-HPLC of KB1 in the presence of native KB1 (nKB1) and observed elution of a single peak, representative of a homogeneous sample (Figure 1 E). Nonetheless, we sought further confirmation of KB1 structural integrity through NMR spectroscopy, including ^1^H chemical shift assignment (Figures S5 and S6) as previously described.[15] Our KB1 chemical shifts align extremely closely with those of nKB1, including a diagnostic ring current-shifted Hβ in residue P6, at −0.17 ppm (Figure S5), and near-identical deviations of Hα chemical shifts from random coil values, indicative of the native structure (Figure 2). We also identified slow-exchanging backbone amide protons in residues C5, C15, T16, S18, V21, C22, T23, R24, L27 and V29 (Figures 2 and S7), as previously observed for nKB1,[15] which definitively confirmed the native fold and characteristic hydrogen bond network.

**Figure 2:**
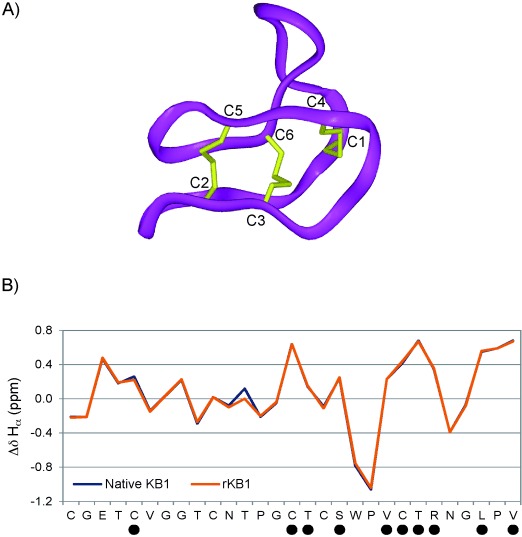
Characterisation of kalata B1 through NMR spectroscopy. A) A ribbon representation of the native KB1 backbone structure, including disulfide-bonded cysteines (in yellow). B) A graphical depiction of the deviation in Hα chemical shift from random coil values for each residue in native KB1 (blue) and our semisynthetic KB1 (orange). • denotes residues in which slow-exchanging amide protons have been identified (Figure S7).

This report represents the first synthesis of a ribosomally derived circular miniprotein without the use of sortase or intein driven cyclization. Each of these existing protocols find wide use but each has limitations. Cyclization using sortase requires incorporation of its five-residue recognition sequence into the circular product, which may not be desirable. Meanwhile, inteins have a tendency to spontaneously undergo in vivo splicing and folding, yielding the product in the bacterial cytoplasm.[16] This phenomenon has facilitated in vivo activity screening of protein libraries,[10] however the inefficiency of the reaction results in low yields of folded protein,[17] and purification of linear precursor for in vitro cyclisation and folding is required to yield comparable quantities of protein to our reported strategy. We therefore believe that following identification of circular protein sequence(s) with a desired activity, a thiol-labile Xaa-Cys might provide a valuable and straightforward strategy for large-scale production, through purification of a stable bacterially derived precursor for chemically mediated in vitro cyclization and folding. Furthermore this method should prove more robust to difficult cases of poor solubility, since the required N-terminal cysteine may potentially also be liberated chemically.[18] Inteins and sortase, on the other hand, require maintenance of tertiary structure for activity and are not compatible with chaotropic agents. We repeated the cyclization of linear KB1 in 6 m guanidinium hydrochloride, and although the reaction proceeded with decreased efficiency; we were pleased to observe majority conversion to the circular product after 72 h (Figure S8).

Due to its cysteine-rich sequence, KB1 represented a challenging molecule for selective C-terminal N→S acyl shift and cyclization. Aside from the C-terminal Gly-Cys, KB1 contains three Val-Cys and two Thr-Cys sequences. Previous studies suggested that cysteine residues preceded by β-branched residues such as valine or threonine are unreactive to intramolecular N→S acyl shift at 45 °C.[7] Similarly, intein-mediated synthesis of KB1 has proved most successful when this glycine, rather than valine or threonine, is positioned adjacent to the active cysteine residue of the intein.[19] Consistent with these observations, we observed selective reactivity at the C terminus.

In conclusion, we report a versatile new strategy for the semi-synthesis of circular proteins that complements the few existing methodologies. The appendage of cysteine to the N and C termini represents a remarkably simple route to molecules for which both in vivo (i.e., endogenous) and established in vitro biosynthesis is rather complex.[20] Here the sequence has been circularly permuted to position cysteine and glycine at the circularization junction. In cases where cysteine is not present, or where it is not present in the desired context (i.e., preceded by Cys/His/Gly) then a non-native Xaa-Cys junction may need to be introduced. However the location of this junction can be specifically designed to avoid active sites or surfaces, or desulfurization of the non-native cysteine could be performed if necessary.[21] That the circular product appears stable to the reverse reaction (i.e., linearization) is an intriguing aspect of this methodology, and is currently under investigation in our laboratory.

## Supporting Information

As a service to our authors and readers, this journal provides supporting information supplied by the authors. Such materials are peer reviewed and may be re-organized for online delivery, but are not copy-edited or typeset. Technical support issues arising from supporting information (other than missing files) should be addressed to the authors.

miscellaneous_information
