# Supplementary material for: Making Ends Meet: Chemically Mediated Circularization of Recombinant Proteins
Source: Chembiochem. 2013 Apr 4;14(7):809–12. doi: 10.1002/cbic.201300105 (PMC4016753; doi:10.1002/cbic.201300105)
Supplement: Supplementary file 1 — miscellaneous_information [file cbic0014-0809-sd1.pdf]

CHEMBIOCHEM

Supporting Information

© Copyright Wiley-VCH Verlag GmbH & Co. KGaA, 69451 Weinheim, 2013

Making Ends Meet: Chemically Mediated Circularization of Recombinant Proteins

Ben Cowper,[a] David J. Craik,[b] and Derek Macmillan*[a]

cbic_201300105_sm_miscellaneous_information.pdf

SUPPLEMENTARY INFORMATION FOR

Chemically-mediated circularisation of recombinant proteins.

Ben Cowper, David J Craik & Derek Macmillan

Supplementary Methods

Supplementary Results

Supplementary Figure 1

Purification of Trx-rKB1 via immobilised metal ion affinity chromatography

Supplementary Figure 2

Factor Xa digestion of Trx-rKB1

Supplementary Figure 3

Trx-rKB1 fragment identification via LC-MS

Supplementary Figure 4

rKB1 circularisation

Supplementary Figure 5

rKB1 ^{1}H chemical shift assignment; ^{1}H-^{1}H TOCSY

Supplementary Figure 6

rKB1 ^{1}H chemical shift assignment; ^{1}H-^{1}H NOESY

Supplementary Figure 7

Amide proton exchange in D_2O

Supplementary Figure 8

rKB1 circularisation under insoluble conditions

SUPPLEMENTARY METHODS

Gene cloning. The DNA sequence encoding kalata-B1 (of sequence; CTCSWPVCTRNGLPVCGETCVGGTCNTPGC-Stop) was amplified via PCR, using primers 5'GGTATTGAGGGTCGCCATATGTGCACCTGTAGCTGGCCGG (forward) and 5'AGAGGAGAGTTAGAGCCTTAACAGCCCGGCGTATTGC (reverse), and cloned into the pET-32 Xa/LIC expression vector via ligation-independent cloning (Novagen). The TEV protease recognition sequence was introduced via mutation of the SGIEGR residue sequence to ENLYFQ. This was carried out via 'inverse' whole-plasmid PCR, using 5'TACTTCCAGTGCACCTGTAGCTGGCCGGTG (forward) and 5'CAGGTTCTCGCCACCACCGGTACCCAGATC (reverse) primers. The resulting linear DNA of approximately 6000 base pairs was then 5' phosphorylated using T4 polynucleotide kinase (New England Biolabs) and ligated using QuickStick DNA ligase (Bioline).

Protein expression & purification. Thioredoxin-kalata B1 (Trx-rKB1) fusion protein was expressed in BL21(DE3) *E. coli* cells via incubation of 500ml LB broth cultures (containing 100 μg/ml ampicillin) at 37°C until an OD_{600} of approximately 0.6, followed by addition of isopropyl-β-D-1-thiogalactoside (Sigma-Aldrich) (1 mM final concentration) and incubation continued for a further 4 hours at 30°C. Cells were harvested via centrifugation, resuspended in lysis buffer (20 mM Tris-HCl pH 8.0, 500 mM NaCl, 5 mM imidazole) and lysed via sonication in the presence of lysozyme (1 mg/ml), 1 mM PMSF, and complete EDTA-free protease inhibitor cocktail (Roche) (one tablet per 1 litre cell culture). Trx-rKB1 was purified through immobilised metal-ion affinity chromatography (IMAC) using Ni-NTA agarose (Qiagen) via gravity-flow, before dialysis against 4 litres of factor Xa cleavage buffer (20 mM Tris pH 8, 100 mM NaCl, 5 mM $CaCl_2$) for 18 hours at 4°C. Linear KB1 precursor peptide was liberated via proteolysis with factor Xa (Merck) (8 units per milligram of Trx-rKB1) at room temperature for 16 hours, or TEV protease (1 mg per 10 mg Trx-rKB1) at 30°C for 4 hours. Both IMAC and proteolysis were monitored via SDS-PAGE and LC-MS. The liberated peptide was purified via reversed phase-high performance liquid chromatography (RP-HPLC).

rKB1 Circularisation. Backbone circularisation was carried out via dissolution of lyophilised linear KB1 precursor peptide in 0.1 M sodium phosphate pH 5.8 (1 mg/ml final concentration), containing 0.5% TCEP and 10 % MESNa. The solution was agitated at 45°C for 48 hours, and the progress of the reaction was verified via LC-MS. The cyclised peptide was isolated via RP-HPLC. Cyclisation under insoluble conditions was carried out in an identical manner, but with inclusion of 6M guanidine hydrochloride, and agitation at 45°C for 72 hours.

Oxidative folding & NMR spectroscopy. Oxidative folding of KB1 was carried out via dissolution of lyophilised cyclic KB1 in 0.1 M ammonium bicarbonate pH 8.5/Isopropanol (50:50) (0.1 mg/ml final concentration). Oxidised glutathione (1 mM) was added and the solution gently stirred at room temperate for 24 hours. Successful oxidation was verified via LC-MS, before purification of the peptide via RP-HPLC. The peptide was then lyophilised and resuspended in 90:10 H_2O/D_2O (pH adjusted to approx. 4 using HCl), and the correct folding was verified via NMR spectroscopy, via acquisition of ^{1}H, ^{1}H-^{1}H TOCSY (mixing time = 100 ms) and ^{1}H-^{1}H-NOESY (mixing time = 200ms) spectra. Spectra were acquired at 25°C on a 600 MHz Bruker DRX spectrometer. Slow-exchanging amides were identified via lyophilisation of the NMR sample, followed by dissolution in 100% D_2O, and acquisition of the ^{1}H NMR spectra at over 24 hours. We define amide signals which remain detectable after five hours in 100% D_2O as slow-exchanging.

Reversed-phase high performance liquid chromatography (RP-HPLC) & Liquid chromatography-mass spectrometry (LC-MS). Preparative RP-HPLC was performed using a Dionex Ultimate 3000 system equipped with a Phenomenex Jupiter 10u Proteo 90Å, C12, 250 x 21.2 mm column. Separations involved a mobile phase of 0.1% TFA (v/v) in water (solvent A)/acetonitrile (solvent B) over a 5-60% acetonitrile gradient. Analytical RP-HPLC was performed using a Dionex Ultimate 3000 system equipped with a Phenomenex SphereClore 5u ODS 80Å, C18, 250 x 4.6 mm column. Separations involved a mobile phase of 0.1% TFA (v/v) in water (solvent A)/acetonitrile (solvent B) over a 5-95% acetonitrile gradient. The sample of native KB1 provided for analytical RP-HPLC comparison was isolated from the plant *Oldenlandia affinis* as previously described[5]. Analytical LC-MS was performed using a Waters Acquity UPLC SQD instrument equipped with an Acquity UPLC BEH 130Å, C18, C18, 2.1 x 50 mm column. Separations involved a mobile phase of 0.1% formic acid (v/v) in water (solvent A)/acetonitrile (solvent B) over a 5-95% acetonitrile gradient.

SDS-PAGE. Extracted samples were denatured via addition to an equal volume of 2X Laemmli loading buffer (containing 10 mM dithiothreitol; DTT) and incubation at 95°C for 2 minutes. Samples were loaded onto 14% bis-Tris polyacrylamide gels, alongside Mark12 molecular weight marker (Invitrogen). Gels were run at 175 V using Novex XCell Sure-Lock apparatus (Invitrogen) and stained using Instant Blue (Triple Red).

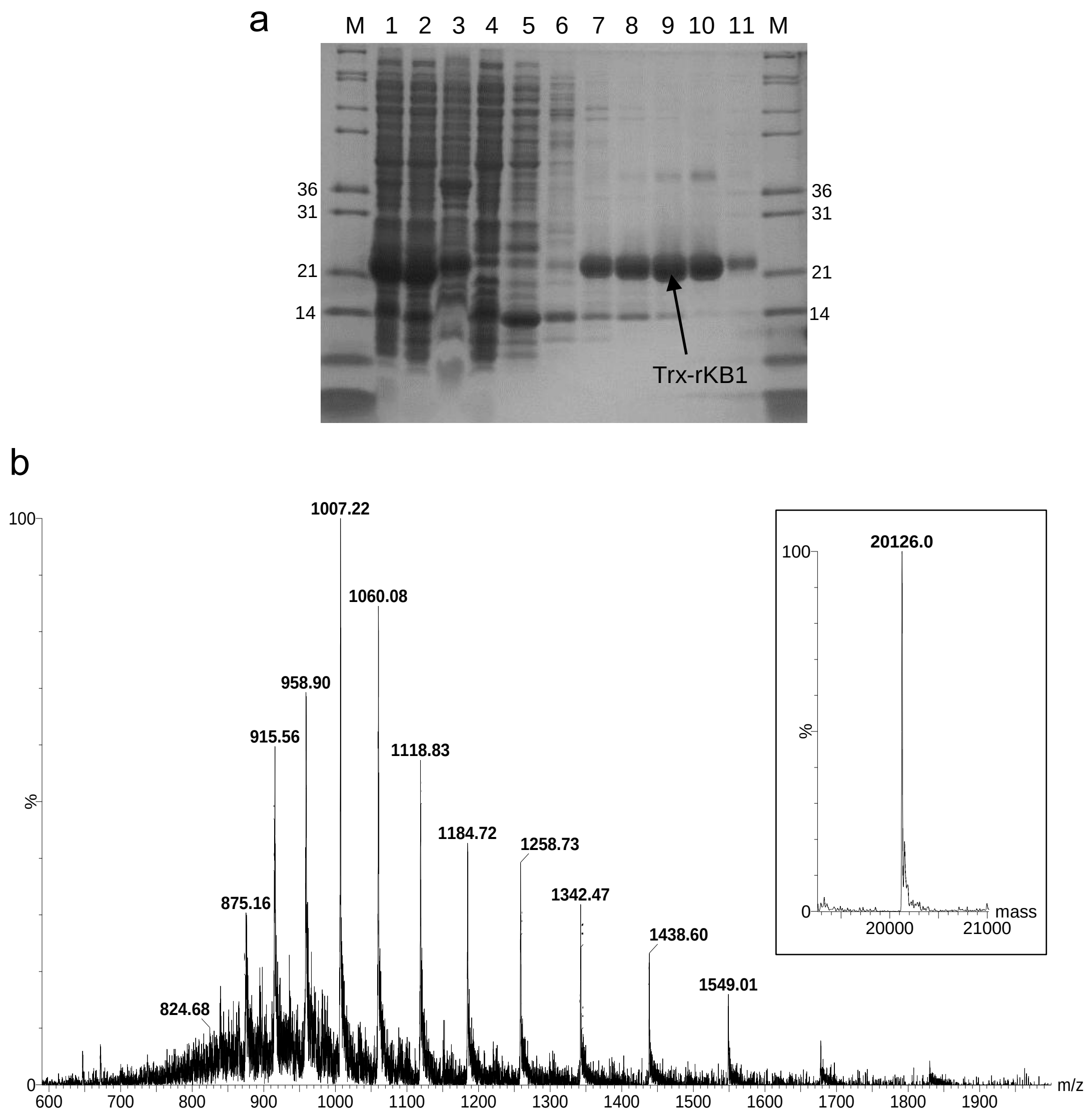

Supplementary Figure 1: purification of Trx-rKB1 via immobilised-metal ion chromatography (IMAC). a) SDS-PAGE analysis of IMAC. M = molecular weight markers; relevant weights are annotated (in kilodaltons). Lane 1 = whole cell lysate, lane 2 = soluble fraction, lane 3 = insoluble fraction, lane 4 = column flow-through, lane 5 = column wash with lysis buffer (5 mM imidazole), lane 6 - column wash with lysis buffer plus 20 mM imidazole, lanes 7-11 = column wash with lysis buffer plus 40, 60, 90, 200 and 500 mM imidazole. The band representing Trx-rKB1 is indicated. **b)** The ES+ mass spectrum of Trx-rKB1. The calculated average mass of the protein is 20127.8 Da.

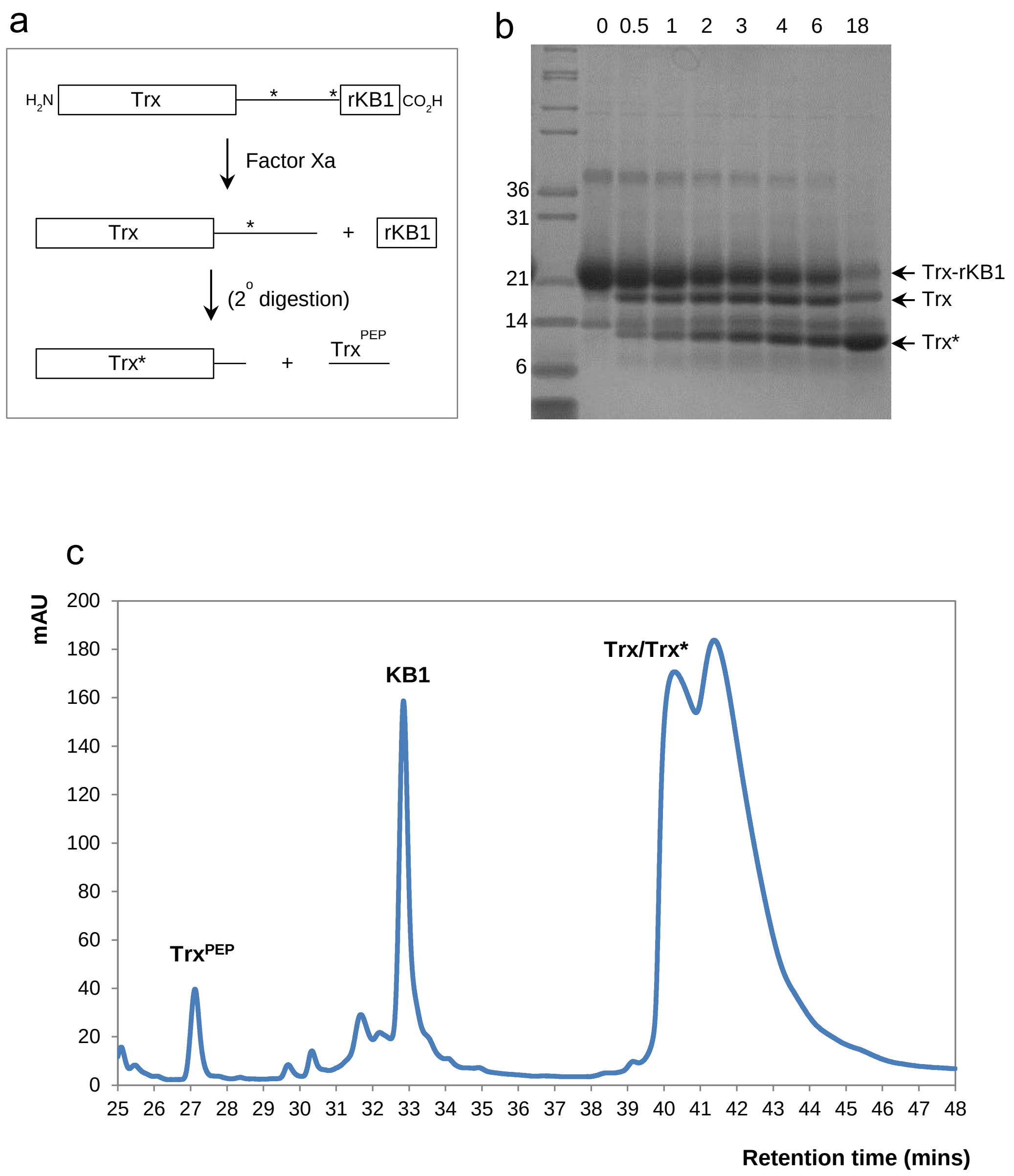

Supplementary Figure 2: Factor Xa digestion of Trx-rKB1 a) A schematic diagram of Trx-rKB1 digestion with factor Xa. Secondary proteolysis at the vector-encoded thrombin recognition site results in digestion of Trx into Trx* and Trx^{PEP} fragments. **b)** SDS-PAGE analysis of factor Xa digestion. Samples were withdrawn at 0, 0.5, 1, 2, 3, 4, 6 and 18 hours following protease addition and run alongside molecular weight markers (relevant weights are annotated). Bands representing Trx-rKB1, Trx and Trx* are annotated. The peptides Trx^{PEP} and rKB1 are not visible on the gel. **c)** RP-HPLC analysis of crude factor Xa digestion. Peaks representing Trx^{PEP}, rKB1 and Trx*/Trx are annotated; identities were verified via LC-MS (Supplementary Figure 3).

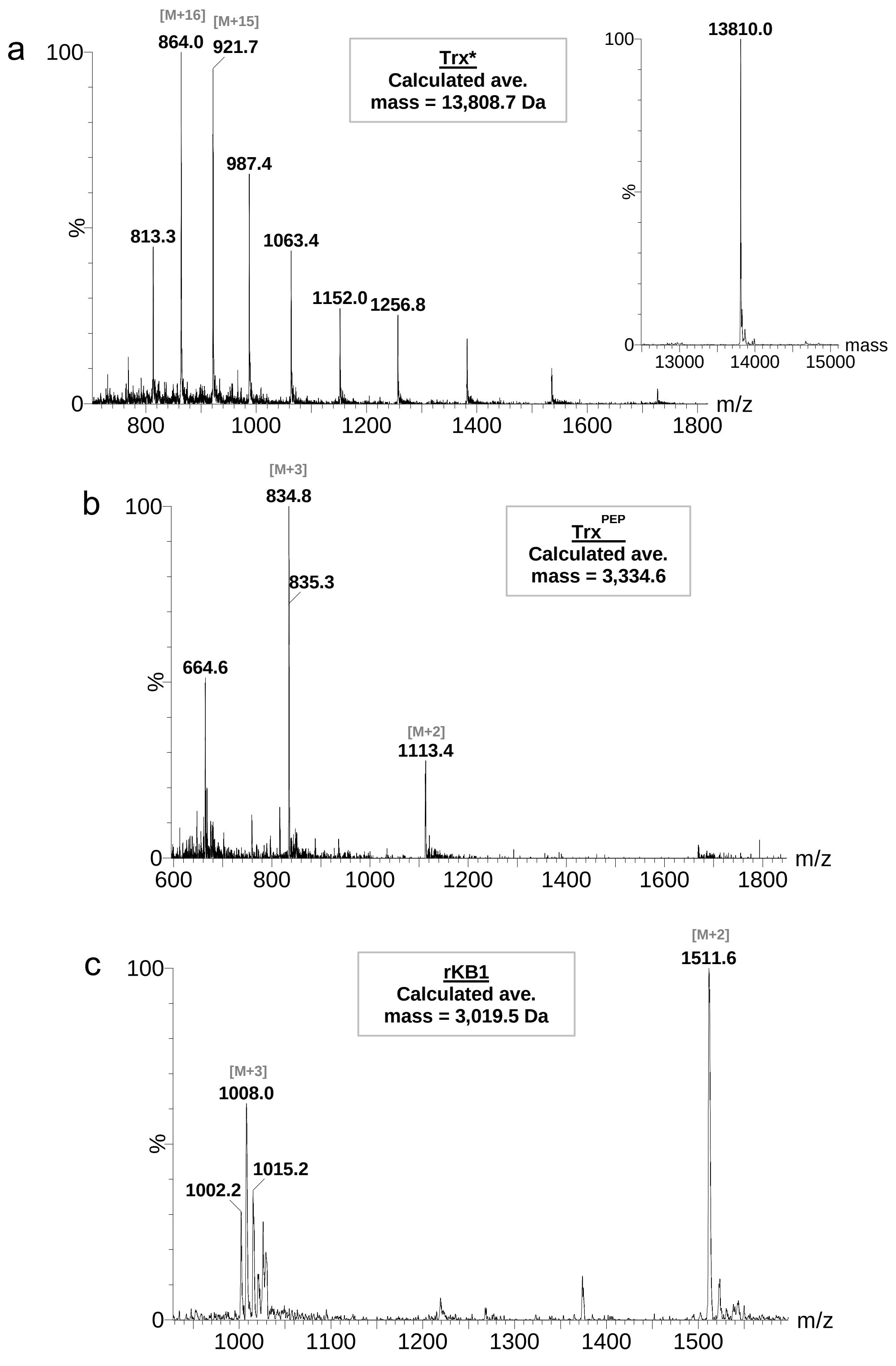

Supplementary Figure 3: Trx-rKB1 fragment identification via LC-MS. Mass spectra of the fragments of factor Xa protease digestion; Trx* (**a**), TrxPEP (**b**), and rKB1 (**c**), obtained via LC-MS analysis of RP-HPLC fractions (Supplementary Figure 2c).

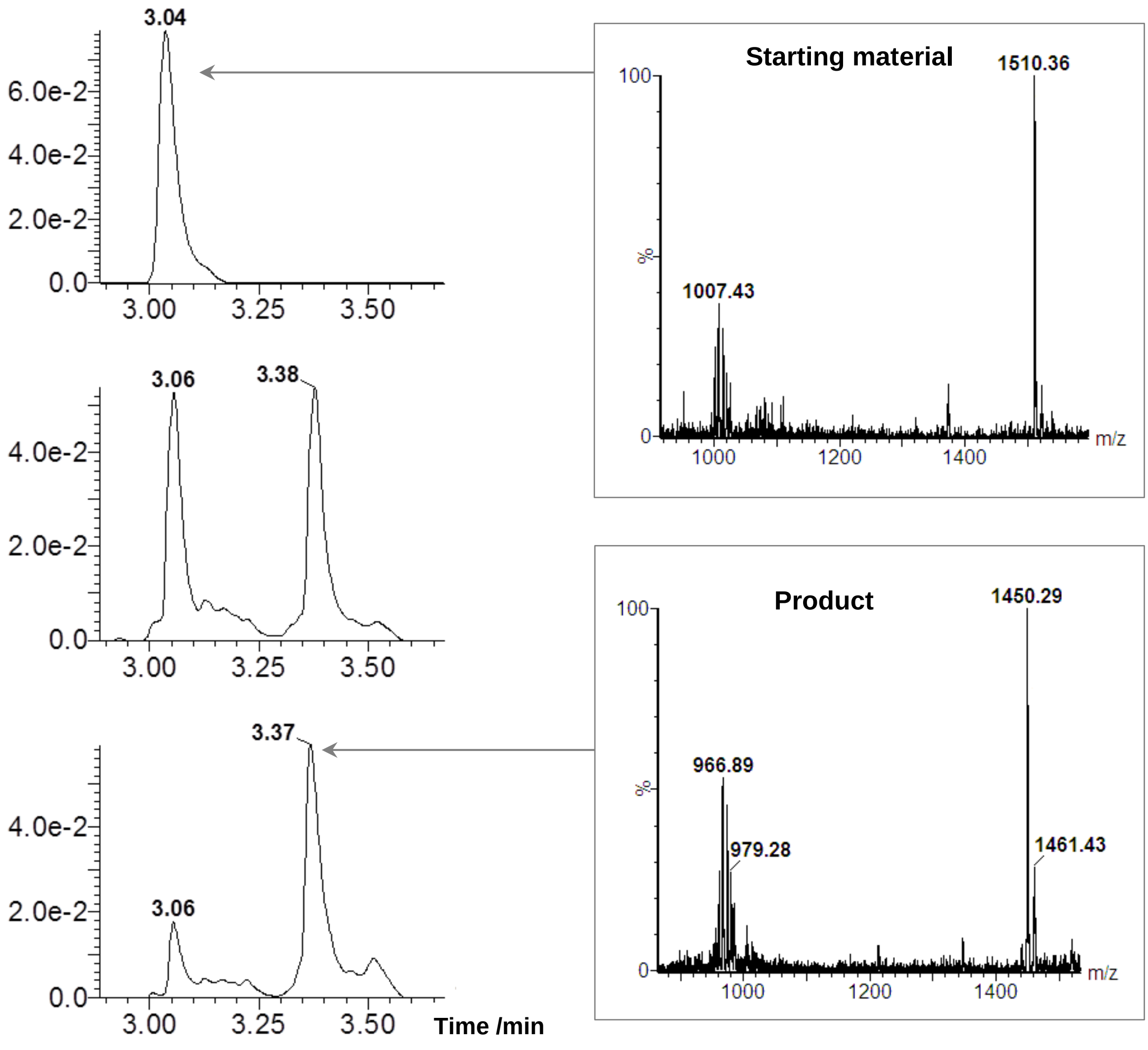

Supplementary figure 4: KB1 circularization. Analysis of KB1 circularization via LC-MS. Samples were analysed at 0, 24 and 48 hours. The different retention times of linear and cyclized KB1 enables clear distinction of starting material and product.

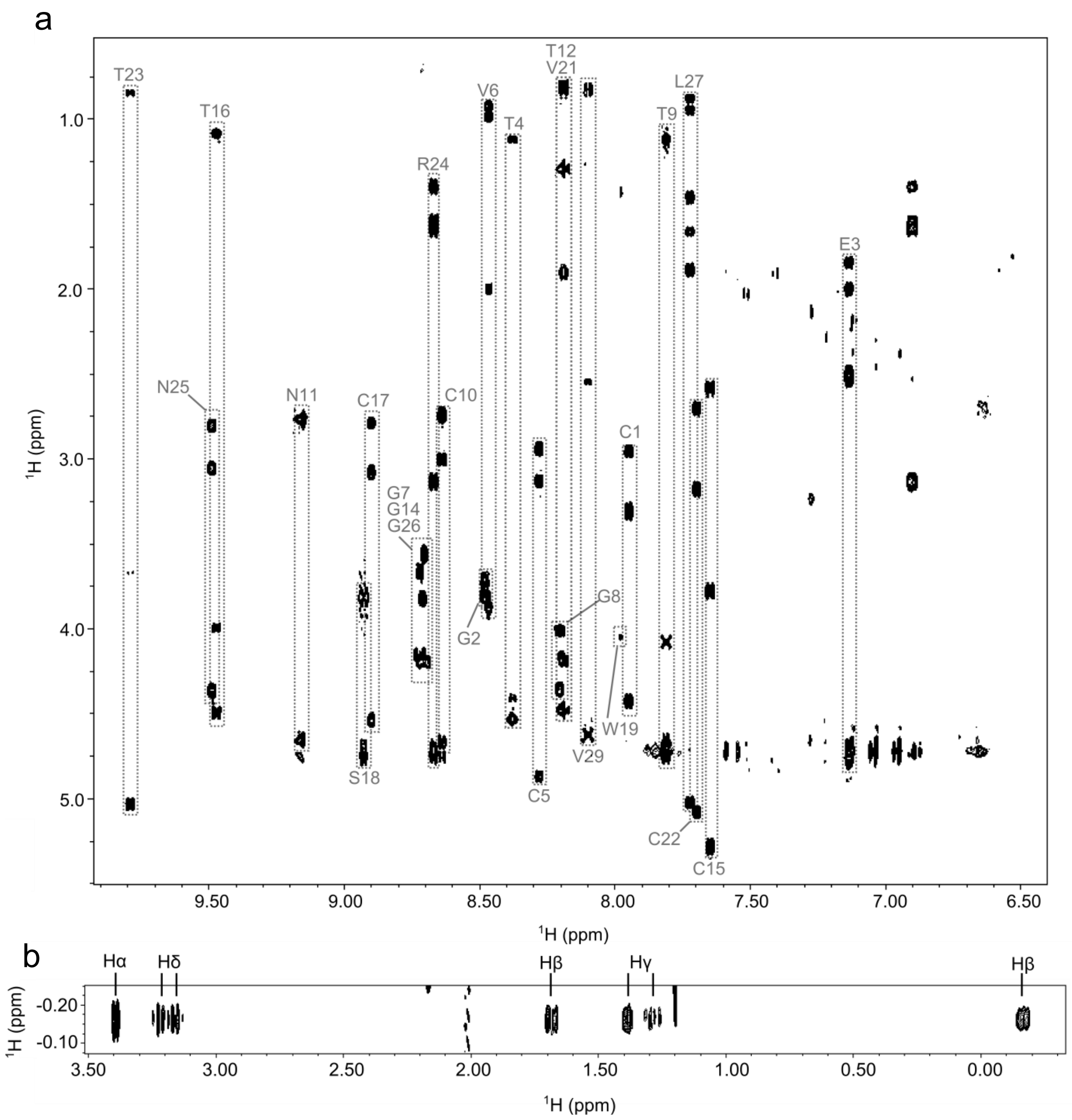

Supplementary Figure 5: rKB1 ^{1}H chemical shift assignment; ^{1}H-^{1}H TOCSY. a) An expansion of the 'amide fingerprint' from the ^{1}H-^{1}H TOCSY spectrum of rKB1, annotated with residue assignments. The chemical shift correlation of the backbone amide with the resonances of the complete associated side-chain gives rise to 'strips' containing all of the resonances of the spin system, facilitating chemical shift assignment. **b)** An expanded section from the ^{1}H-^{1}H TOCSY spectrum of rKB1, displaying the side-chain chemical shifts of residue P6. The Hβ chemical shift below 0.00ppm is diagnostic of native KB1 structure.

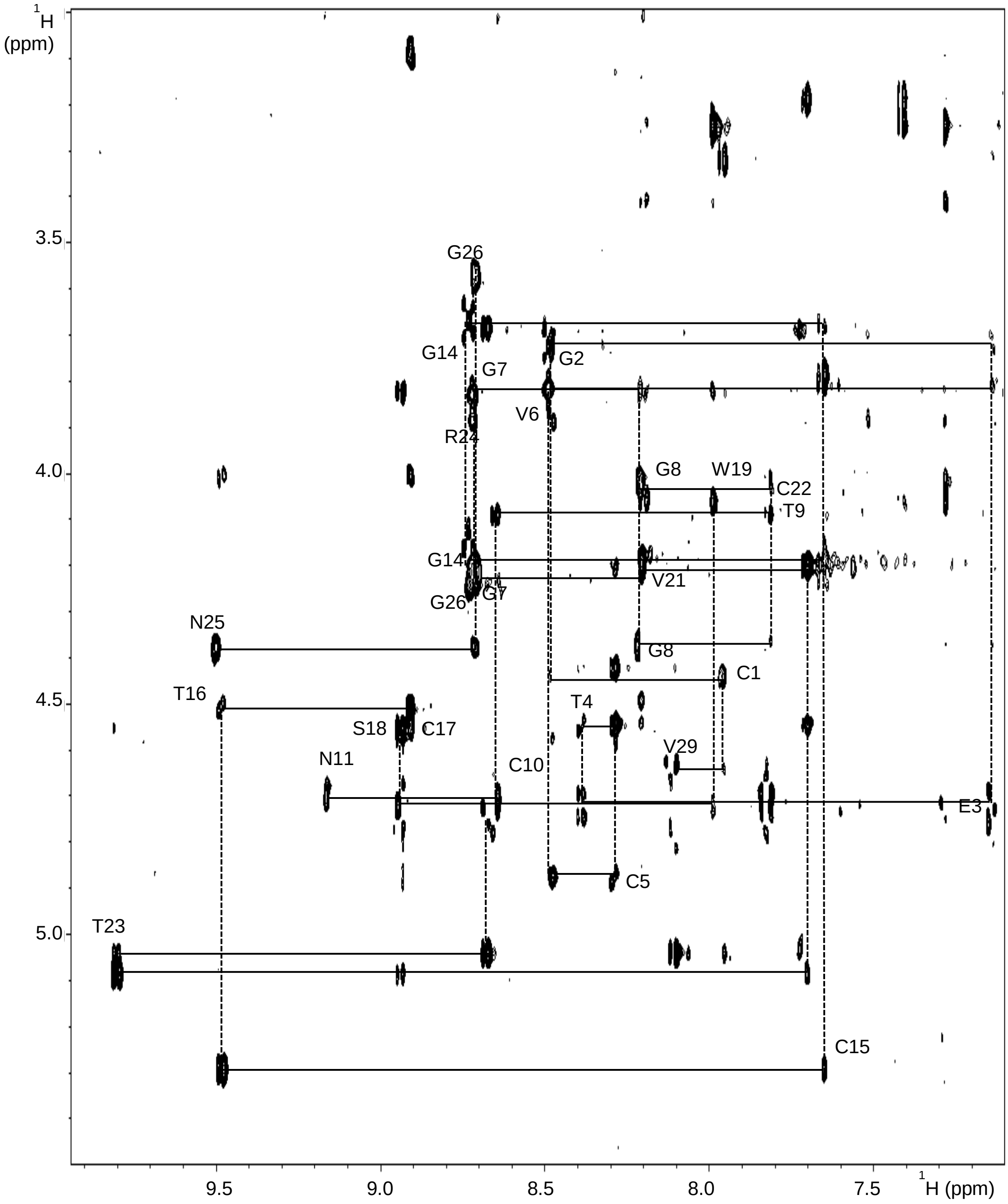

Supplementary Figure 6: KB1 ^{1}H chemical shift assignment; ^{1}H-^{1}H NOESY. An expanded section of the ^{1}H-^{1}H NOESY spectrum of rKB1. Cross-peaks between Hα (residue i) and HN (residue i+1) chemical shifts are annotated.

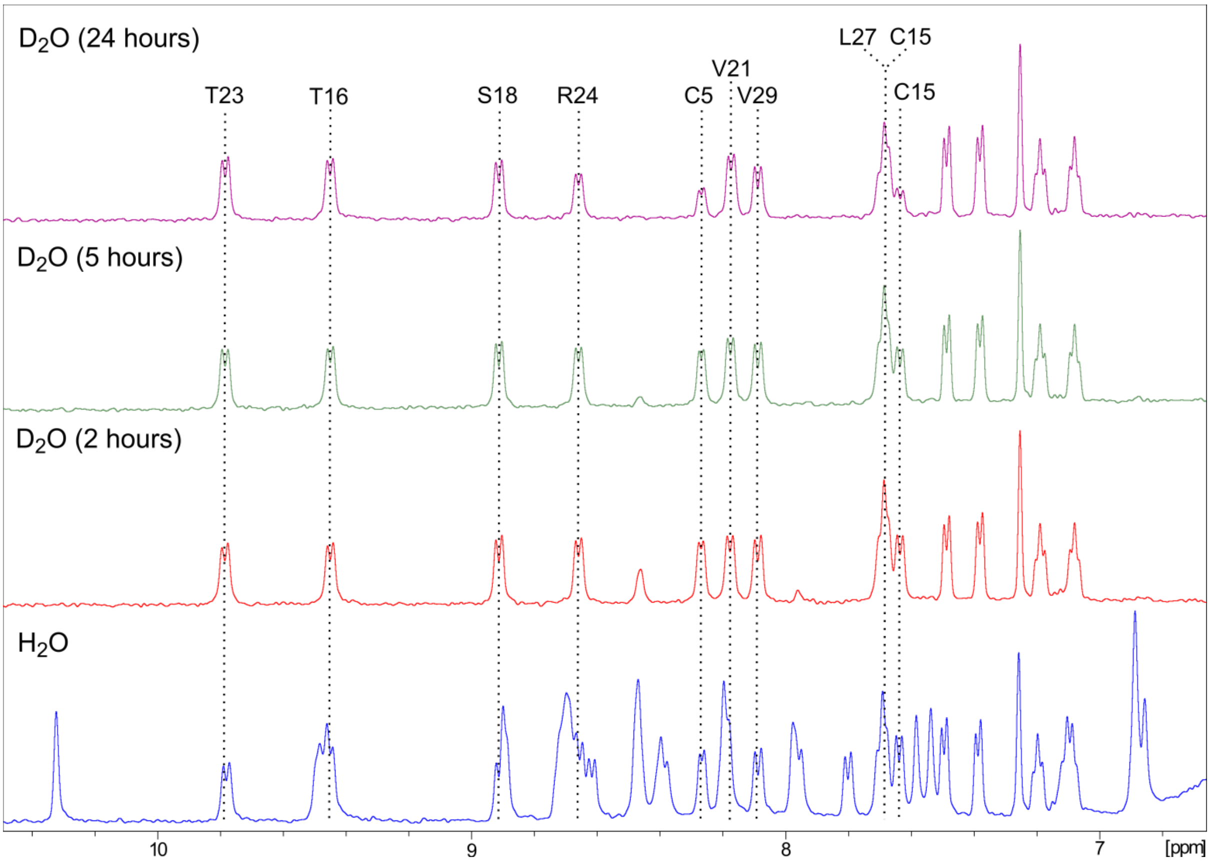

Supplementary Figure 7: Amide-proton exchange in D_2O. 1D NMR spectra of KB1 in H_2O (blue), and 2 hours (red), 5 hours (green) and 24 hours following dissolution of lyophilised peptide in 100% D_2O. Amide proton resonances which are visible in the spectrum following 5 hours in D_2O are classified as slow-exchanging.

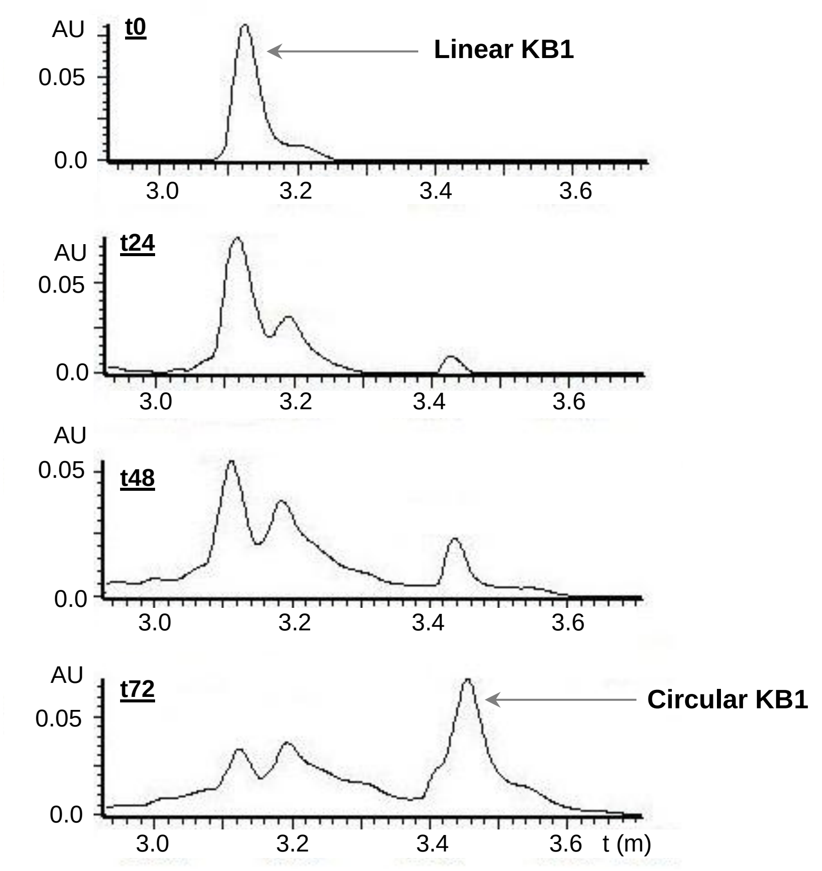

Supplementary figure 8: KB1 circularisation under insoluble conditions. Analysis of KB1 circularisation in 6M guanidine hydrochloride via LC-MS. Samples were analysed at 0, 24, 48 and 72 hours.
